# Co-Design and Refinement of Curriculum-Based Foodbot Factory Intervention to Support Elementary School Nutrition Education

**DOI:** 10.3390/nu16213769

**Published:** 2024-11-02

**Authors:** Jacqueline Marie Brown, Nicholas Rita, Beatriz Franco-Arellano, Ann LeSage, JoAnne Arcand

**Affiliations:** 1 Faculty of Health Sciences, Ontario Tech University, Oshawa, ON L1G 0C5, Canada; 2 Mitch and Leslie Frazer Faculty of Education, Ontario Tech University, Oshawa, ON L1G 0C5, Canada

**Keywords:** nutrition education, serious games, children, food literacy, school nutrition intervention, co-design

## Abstract

Background/Objectives: School-based nutrition education interventions can support the development of children’s food literacy and healthy eating habits. The Foodbot Factory serious game was developed to support school nutrition education based on Canada’s Food Guide and Ontario curriculum. The objective of this research was to refine the Foodbot Factory intervention to include curriculum-based lesson plans that had a high-level of acceptability by stakeholders to support implementation by teachers in classrooms. Methods: A co-design approach was used to engage teacher and dietitian stakeholders in developing five lesson plans for the intervention, who contributed to creating the intervention content in three stages. The stages included reviewing and providing feedback on the initial draft of the lesson plans, participating in facilitated discussion rounds to come to a consensus on the changes required, and completing a final review of the intervention’s acceptability. Qualitative data included notes on the lesson plans and recordings from meetings that were analyzed thematically. Results: During the first co-design stage, major revisions were suggested for two-fifths of the lessons by stakeholders. Further stakeholder suggestions were discussed and integrated into the intervention from facilitated discussions, improving the lesson plan content and intervention feasibility. All stakeholders agreed that the final version of the intervention was acceptable and would support classroom nutrition education. Five lesson plans were created and compiled into a unit plan, containing additional teaching resources, to support nutrition education with Foodbot Factory. Conclusions: The co-design process greatly improved the Foodbot Factory intervention and its feasibility for classroom implementation. Including diverse stakeholder perspectives led to unique and different insights to improve the intervention.

## 1. Introduction

Food literacy encompasses the interconnected knowledge, attitudes, and skills that influence dietary behaviours [1]. Among children, higher levels of food literacy are associated with improved diet quality [2]. Unfortunately, in Canada, the average child’s diet does not meet the recommendations for vegetables, fruit, and whole grain foods and exceeds the recommended intake for nutrients of public health concern, such as sodium, sugar, and saturated fat [3,4]. Thus, improving children’s food literacy has been identified as a public health priority in Canada, and accessible, evidence-based interventions are required to achieve this aim [5].

School-based nutrition education can be an effective and equitable way of improving children’s food literacy and, subsequently, their healthy eating habits [6,7]. Nutrition is a mandatory component of the health education curriculum in primary and secondary schools in Canada, as well as many other schools internationally. However, teachers report several barriers to implementing nutrition education in their classrooms. These barriers include a lack of training and time dedicated to nutrition education and low self-confidence in teaching nutrition [8]. Canadian teachers have reported relying on pre-existing resources for nutrition education, such as those sourced online or from colleagues, but there is a paucity of high-quality, evidence-based resources designed specifically for elementary school classrooms [9]. Nutrition education interventions that utilize technology can help address these barriers, as technology is highly accessible in Canadian classrooms and often time-efficient to implement [10]. Serious games, which are games designed specifically for education, are emerging as a leading educational tool due to their ability to provide an experiential learning approach and engage learners [11]. A systematic review of serious games designed for nutrition demonstrated that these interventions can improve nutrition knowledge and dietary behaviours [12]. Nevertheless, further research is required to specifically evaluate how serious games can be implemented in the classroom context to improve curriculum-based nutrition education.

To support teachers in teaching nutrition, our research team iteratively developed and evaluated the Foodbot Factory mobile serious game [13,14]. The game’s content aligns with health and physical activity curricula across Canadian provinces (Grades 4 or 5) and Canada’s Food Guide (CFG). It has been shown to significantly improve children’s nutrition knowledge, which is a core component of food literacy [15]. However, it has not yet been evaluated in its intended classroom environment. Technology-based educational tools alone are usually insufficient for teachers to use in their classrooms; teachers report needing additional information and resources to support their implementation of new digital learning tools [16]. Before evaluating the Foodbot Factory intervention in classrooms as part of the nutrition education curriculum, refinement of the intervention to include lesson plans and resources to support teachers in implementing the serious game in real-world classrooms was needed based on the needs and opinions of stakeholders through a co-design approach [16,17]. A co-design approach was chosen to include the perspectives of end users of the intervention in the design with the goal of supporting future implementation [18]. The objective of this paper is to describe the co-design approach taken with multiple stakeholders to refine the Foodbot Factory nutrition education intervention and the resulting intervention that will be evaluated in classrooms and made available for teachers across Canada.

## 2. Materials and Methods

A co-design participatory approach was used to develop and refine the Foodbot Factory lesson plans to complement the existing serious game, ensuring high acceptability, suitability, and feasibility among end users between April and November 2022. This collaborative process involved multiple parties, including a research team consisting of a PhD student and postdoctoral fellow in nutrition, a hired certified teacher, and two faculty members in Health Sciences and Education. The external stakeholders that engaged in the co-design process included three certified elementary teachers and two registered dietitians working in school nutrition and public health, who provided unique disciplinary perspectives [19]. Stakeholders participated in the design of the intervention in three stages: critically reviewing and revising the initial drafts of the lesson plans, participating in facilitated discussion rounds, and completing a final review and evaluation of the acceptability of the lesson plans. Since stakeholders participated in the co-design process as equal members and no identifying information was collected from them, REB approval was not sought for this study.

### 2.1. Refinement of the Foodbot Factory Intervention to Include Lesson Plans

Prior to refining the Foodbot Factory intervention to include lesson plans, the research team established guiding principles: The intervention would consist of five daily lessons delivered over one week, with each lesson being approximately 35–40 min. We chose these timings from the outset of the intervention refinement process to correspond to the typical length of an instructional unit in an elementary school classroom, which would support real-world implementation. We also pre-determined that classrooms would play one module of the Foodbot Factory serious game per lesson and that the daily lesson topics and learning objectives would correspond to the game’s learning modules and content (Drinks, Whole Grain Foods, Vegetables and Fruit, Animal Protein Foods, Plant Protein Foods). As the Foodbot Factory serious game utilizes experiential learning theory [20], we pre-determined that other learning activities developed as part of the Foodbot Factory lesson plans would be grounded in constructivist learning theory to help learners connect their new knowledge about nutrition to their lived experiences, culture, food traditions, and pre-existing knowledge [21].

The lesson plans were based on CFG and the Ontario Health and Physical Education curriculum for Grades 4 and 5 (ages 8–12) and guided by the content, flow, and organization of the Foodbot Factory serious game [22,23]. The content covered in the Ontario curriculum for this age group, which was last updated in 2019, is similar to the curriculum used in other Canadian provinces. The curriculum covers three specific learning expectations at this stage: identify food and beverage sources of key nutrients and describe how they influence health; identify personal eating habits; and identify ways of promoting healthier eating [23]. As teachers have limited time to provide nutrition education, the topics of the intervention were restricted to covering the curriculum learning expectations to enhance usability, acceptability, and future implementation. During the refinement of the intervention, the team made no changes to the Foodbot Factory serious game itself.

### 2.2. Co-Design and Evaluation of the Nutrition Education Intervention

**Figure 1:**
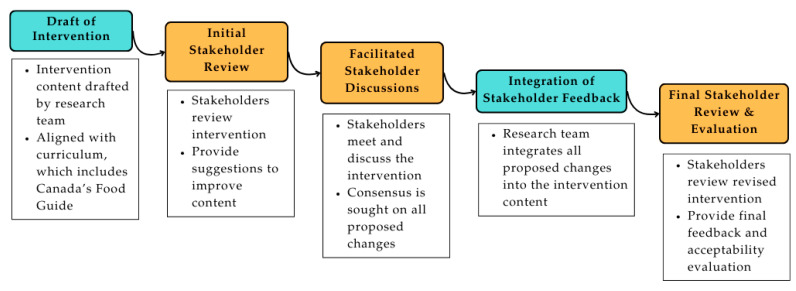
Co-design of the Foodbot Factory intervention with stakeholders.

The initial drafts of the lesson plans were written by the student carrying out a PhD in nutrition and the hired certified teacher, and these drafts were critically reviewed by the other members of the research team. We recruited stakeholders to participate through our professional networks, and they were offered a CAD 100 gift card for a retailer of their choice as compensation for their time and expertise. The stakeholders initially provided input by actively editing and commenting on the drafts and identifying components of the lesson plans that required significant changes. The teacher stakeholders participated in two 60 min meetings, while the dietitian stakeholders participated in one 120 min meeting. The goal of these meetings was to reach a consensus on the proposed changes by reviewing feedback and discussing new ideas to improve the lesson plans. Each proposed change was reflected upon using facilitated discussion rounds, where each stakeholder shared their thoughts and then consensus on the change was sought [24]. If disagreement arose regarding a proposed change, the change underwent further discussion until a consensus was achieved. After the meetings, the research team modified the lesson plans to incorporate the agreed-upon changes. The revised lesson plans were re-circulated to the stakeholders for final review and evaluated using probing questions. The co-design process is illustrated in Figure 1.

### 2.3. Data Collection and Analysis

Qualitative data, collected from the revisions made to the lesson plan documents, meeting notes, and open-ended question responses, were analyzed thematically. Each stakeholder independently answered a set of final probing questions on the lesson plans, developed using the Theoretical Framework of Acceptability, indicating their views on the intervention’s overall acceptability, perceived effectiveness, intervention coherence, self-efficacy, and burden to implement in classrooms [25].

## 3. Results

The research team created a draft of five lesson plans to complement and support the implementation of the Foodbot Factory serious game. After reviewing the first draft, stakeholders (n = 5) suggested major revisions to two of the five lesson plans. One major revision was to include a greater variety of food choices in the Animal Protein Foods lesson. Another major revision suggested was to combine the separate Animal and Plant Protein Foods lessons into one lesson. However, this suggestion could not be accommodated because of the modular structure of the Foodbot Factory serious game and the guiding principle that one module would be played per day. Stakeholders also suggested ideas for improving the adaptability of the lesson plans to more easily integrate different cultural eating patterns and student learning needs. Here, they proposed new learning activities for students and suggested additional background content on nutrition topics for teachers. The teachers contributed ideas to improve the feasibility of implementing the intervention, such as adding information on lesson preparation times and resources for teachers to consider when teaching nutrition. Meanwhile, the dietitians provided suggestions on educational content suitability, emphasized the importance of using neutral language for food, and ensured the language used was consistent with CFG.

The final Foodbot Factory lesson plans were compiled into a unit plan, which is a document that presents a series of learning activities focused on achieving specific learning goals. Through the co-design process, we learned that teachers require more overarching information on how to teach the nutrition unit, beyond the content contained in the five daily nutrition education lessons. Given the novelty of Foodbot Factory, they also required more information about the serious game itself and its features, to promote future implementation. Therefore, the research team and stakeholders developed a repository of supporting information within the Foodbot Factory intervention unit plan, including guidance on teaching nutrition (e.g., resources on overarching philosophies when teaching nutrition to children), accommodations for students, references to the curriculum, and a summary of Foodbot Factory’s accessibility features (Table 1).

The unit plan contained five lessons using Foodbot Factory, with the intention that one lesson would be provided per day over the course of a week. Each daily lesson plan was aligned with the three-phase lesson structure, consisting of *Phase I: Getting Started*, *Phase II: Exploration*, and *Phase III: Consolidation* [26]. This structure was chosen because it is considered effective and is commonly used by teachers. In *Phase I: Getting Started*, learning activities introduce students to the daily topic via discussion questions, videos, and activity sheets. In *Phase II: Exploration*, teacher-facing instructions have students play the corresponding daily learning module in the Foodbot Factory serious game. In *Phase III: Consolidation*, learning activities are used to help students connect new knowledge from the game to their previous knowledge and lived experience, such as discussion questions and teacher-led activities. Table 2 presents a summary of the lesson plan topics, activities, and learning objectives for the Foodbot Factory intervention.

In the final evaluation of the lesson plans, all stakeholders (n = 5) reported that the lesson plans were acceptable and would be effective in supporting student learning. They found the intervention materials cohesive and they believed teachers would feel confident implementing the intervention. Two stakeholders reported concerns on the time burden for implementation, as teachers would need to take more time to learn how to use the serious game prior to implementation compared to a non-technology-based learning tool. However, they also commented that the extra time taken for teachers would be worthwhile to provide a more interactive, engaging, and comprehensive learning experience for students.

## 4. Discussion

In our research, we co-designed curriculum-based nutrition education lesson plans and supplementary unit plan resources to support the implementation of the Foodbot Factory serious game in elementary school classrooms. This pragmatic intervention component, developed with dietitians and teachers, is crucial to enable the use of the Foodbot Factory intervention in real-world classrooms [16]. It was also necessary to prepare for future research that will evaluate Foodbot Factory in classrooms as part of the nutrition education curriculum. Schools require tailored and specific implementation strategies for nutrition education, and there is a critical need for structured resources and lesson plans to support school nutrition education, as we have developed in this research. This need is evidenced by Health Canada’s recent release of a toolkit for educators to use when teaching with CFG, an important resource that can be used to support nutrition education and is part of the provincial nutrition curriculum nationwide [27]. However, the CFG toolkit for educators does not include hands-on resources that are tailored and adaptable for teachers to use in the classroom, as we have now created. The Foodbot Factory lesson plans prioritize adaptability and accessibility to diverse cultures and learning needs, which is a key implementation strategy for classroom-based interventions and particularly important for technology-based interventions with set features and content [16]. We also tailored the Foodbot Factory unit and lesson plan content to address barriers teachers have reported in nutrition education. This includes providing resources on how to teach nutrition to improve teacher’s self-efficacy for nutrition education and creating time-efficient lessons to ensure teachers can cover the content in the minimal time that is often allocated for nutrition [8,16].

This research meaningfully engaged stakeholders in the development and refinement of the intervention, which is increasingly recommended for intervention design [28,29]. By centering end users in the intervention’s development, co-design can lead to many benefits. These include improving the cultural and contextual fit of an intervention within a given setting, bringing diverse perspectives into the development process so that an intervention meets the needs of multiple stakeholders, and minimizing barriers and bottlenecks to real-word implementation since these are shared by stakeholders throughout the design process. While further evidence is needed to establish the efficacy of co-designing an intervention compared to traditional intervention development methods, involving stakeholders in the co-design process can lead to a more acceptable and suitable intervention [30]. For example, in the development of a health promotion intervention for adolescents, co-design with the target audience resulted in the identification of contextual influences on the intervention topic that otherwise may not have been included [31]. Similarly, another study, which used co-design for an intervention targeting fathers and their child’s feeding behaviours, highlighted the importance of being able to tailor content to an individual’s needs [32]. In our co-design process, we observed similar benefits, as the ability to tailor nutrition lessons to meet the unique needs of each classroom emerged with stakeholders, demonstrating the need for nutrition interventions to be adaptable to different cultures, contexts, and needs. Our study also benefited from having stakeholder representation from both dietetics and education professionals, a collaborative approach leading to the identification of new and different ways to improve the intervention and address different barriers to its implementation. We found that the input from teachers focused on improving the feasibility of classroom implementation, while the input from dietitians led to enhancing the suitability of the nutrition content. The choice to include multiple different professions in the co-design process is supported by other studies, where it was felt this approach led to the identification of a greater breadth of ideas that can support an intervention’s success [33].

A key strength of this study was involving an engaged committee of stakeholders from different disciplines to contribute to the intervention’s co-design in an iterative process. A possible limitation of this research is that we engaged stakeholders in three stages of co-design, whereas other co-design research studies have provided more varied opportunities for stakeholder input (e.g., workshops and surveys) [32,33]. Including additional stages of co-design and ways to provide feedback may have led to the identification of additional ways to improve the intervention that were not captured. We also did not include children, the intervention’s target audience, in the lesson plan co-design process. However, we have previously conducted iterative user testing with children to ensure the acceptability of the Foodbot Factory serious game [13]. This co-design research is part of a larger programme of research seeking to further our understanding of how serious games can be leveraged to support nutrition education for both children and teachers. The research described in this manuscript was essential to ensuring the Foodbot Factory intervention would be suitable for classroom use and meet the needs of stakeholders. Based on existing research demonstrating the effectiveness of both nutrition-focused serious games and curriculum-based nutrition education interventions, we believe that the Foodbot Factory intervention, which combines these approaches, will be a useful and beneficial tool for nutrition education [12,34]. Currently, the intervention is being evaluated in a cluster-randomized controlled trial, which will determine the efficacy of the Foodbot Factory intervention in improving children’s nutrition knowledge, attitudes, and behaviours [35]. Data collected from children and teachers in this ongoing study will be used to further refine and improve the intervention.

## 5. Conclusions

This paper describes the refinement of a curriculum-based nutrition education intervention, Foodbot Factory, using a co-design approach with dietitian and teacher stakeholders. Our findings led to the Foodbot Factory intervention having improved acceptability, feasibility, and suitability among those who will implement the intervention in their work practices. Furthermore, this research adds to the growing body of research on co-designing interventions and the importance of involving stakeholder perspectives [30]. Future qualitative research will explore teachers’ perceived barriers and facilitators to using Foodbot Factory in classrooms to inform future strategies to support implementation scalability and sustainability in Canadian classrooms.

## Figures and Tables

**Table 1:** Description of the components of the Foodbot Factory unit and lesson plans.

Unit Plan Component	Description
Guidance on Discussing Nutrition with Students	Presents an overarching philosophy for talking about food and nutrition throughout every lesson. This section emphasizes Taking a positive approach to discussing foods by encouraging curiosity and not labelling foods as “good” or “bad”. Connecting nutrition to the real world and recognizing that children may not control what they eat. That teachers reflect on their own views about nutrition and how this may influence their teaching practice. That teachers utilize links to other resources provided to learn more about nutrition and teaching nutrition to students.
Teaching with Foodbot Factory	Provides an overview of the Foodbot Factory serious game, lesson plans and the content in each lesson.
Curriculum Connections	References the Ontario Physical Health and Education curriculum expectations, Strands D1,1, D2.1, and D3.1 [14].
Foodbot Factory Accessibility Features	Describes the accessibility features of the Foodbot Factory serious game (e.g., sounds, voices, large text).
Accommodations for Students	Suggests different ways teachers can work with their classroom to accommodate and modify the lessons to their unique learning needs. Emphasis is given to differentiating class instructions for students and providing flexible ways for students to demonstrate their learning.
Lesson Plan Component *	Description
Lesson Overview	Summarizes the lesson’s topic and content.
Teacher Considerations for the Lesson	Provides teachers with guidance on tailoring and adapting the lesson for their unique group of students. Each lesson provides considerations on Food accessibility and affordability; Integrating cultural foods and dishes, including those for Indigenous populations, with examples provided; Information on some common dietary restrictions (e.g., overview of gluten intolerance and celiac disease in the Whole Grain Foods lesson).
Learning Goals and Success Criteria	Describes the learning goals (i.e., what students will be able to do by the end of the lesson) and success criteria (i.e., how students will be able to demonstrate their knowledge) for the lesson.
Key Messages for Students	Summarizes the key nutrition messages for the lesson.
Scaffolding via Gradual Release of Responsibility and Feedback	Describes each component of the three-phase lesson (Phase I: Getting Started, Phase II: Exploration, and Phase III: Consolidation), with instructions on how to guide students through the lesson. Includes guidance on assessments of student learning and what is required to prepare the lessons (e.g., setting up a slideshow, printing handouts).

* Available for each of the five nutrition lessons.

**Table 2:** Foodbot Factory intervention learning topics, activities, and objectives.

Daily Topic	Learning Objectives	Phase 1: Getting Started	Phase 2: Exploration	Phase 3: Consolidation
		Introductory Activities and Set Expectations (~10 min)	Main Lesson Activity (~10–15 min)	Summarize and Review Lesson (~10 min)
Day 1: Drinks	Evaluate the best beverage choice for staying hydrated. Distinguish the health impacts of different drink choices (e.g., water, milk, sugary drinks). Recall different types of sugary drinks.	Introductory slideshow on food and drinks Teacher-led class activity Class discussion	Play “Drinks” module in Foodbot Factory serious game	Complete Phase 1 class activity, adding to it based on what was learned Class discussion
Day 2: Whole Grain Foods	Identify the nutritional difference between whole grain foods and refined grain foods. Explain how whole grain foods and fibre impact our health. Determine how grains can be integrated into our daily meals.	Introductory slideshow Class discussion	Play “Whole Grain Foods” module in Foodbot Factory serious game	Small-group activity and discuss answers Class discussion
Day 3: Vegetables and Fruit	Describe why vegetables and fruit are important for our health. Explain the importance of eating a variety of veggies and fruit. Identify and describe why highly processed foods should be limited. Demonstrate how much of a plate should be dedicated to vegetables and fruit.	Introductory slideshow Complete activity sheet on vegetables and fruit independently Class discussion	Play “Vegetables and Fruit” module in Foodbot Factory serious game	Complete Phase 1 activity sheet, adding to it based on what was learned Class discussion
Day 4: Animal Protein	Explain the importance of eating a variety of protein foods. Recognize the health impacts of dietary fats and sodium. Explain why processed meats should be consumed less often.	Introductory slideshow Class discussion	Play “Animal Protein” module in Foodbot Factory serious game	Teacher-led class activity Class discussion
Day 5: Plant Protein	Identify a variety of plant protein foods. Explain the health and environmental benefits of choosing plant protein foods. Explain why unsweetened soy milk is a protein food.	Introductory slideshow Class discussion	Play “Plant Protein” module in Foodbot Factory serious game	Teacher-led class activity Class discussion

## Data Availability

The original contributions presented in this study are included in the article. Further inquiries can be directed to the corresponding author.
